# Fearful or functional - a cross-sectional survey of the concepts of childhood fever among German and Turkish mothers in Germany

**DOI:** 10.1186/1471-2431-11-41

**Published:** 2011-05-23

**Authors:** Thorsten Langer, Miriam Pfeifer, Aynur Soenmez, Bilge Tarhan, Elke Jeschke, Thomas Ostermann

**Affiliations:** 1 Institute of General Practice and Family Medicine, Witten/Herdecke University, Witten, Germany; 2 Children's Hospital, HELIOS Klinikum, Wuppertal, Germany; 3 Institute of Nursing Science, Witten/Herdecke University, Witten, Germany; 4 Havelhoehe Research Institute, Berlin, Germany; 5 Center for Integrative Medicine, Witten/Herdecke University, Herdecke, Germany

## Abstract

**Background:**

Fever is one of the most common presenting complaints in paediatrics and general practice. In the majority of cases nothing harmful is diagnosed. However, the subjective meaning of fever often varies between doctors and parents. Knowledge of the parents' concept of fever may help tailor counselling to their needs.

In this study we determine 1) the influence of socio-economic status and cultural background on two concepts of fever which we labelled "functional" and "fearful", each representing typical experiences of mothers, and 2) the actions taken by the mothers related to these concepts.

**Methods:**

A standardized interview study was conducted among German and Turkish mothers in Germany in 2009. The questionnaire consisted of 36 questions and 205 items. Interviews were conducted in 16 private practices of paediatricians and 2 paediatric emergency departments in an urban region of Germany. The two fever concepts were represented in 6 statements that could be rated with a six-point Likert scale. The association of the socio-economic status and the cultural background with one of the fever concepts was determined by a multiple logistic regression.

**Results:**

A total of 338 mothers (49% with a Turkish background) completed the interview (response rate 92%). The average age of mothers with a German background was higher (34.1 years vs. 32.0 years, p = 0.0001). Mothers with a Turkish background were more likely to relate to the concept "fearful" [adjusted Odds Ratio (AOR) 1.99; confidence interval (CI) 1.16-3.44]. Mothers with a middle or high socio-economic status were more likely to respond to the concept "functional" [middle: AOR, 0.53; CI, 0.30-0.92; high: AOR, 0.44; CI, 0.21-0.95].

Mothers adhering to the concept "fearful" more often gave acetaminophen before the recommended interval of 6 hours (46.8% vs. 31.3%, p = 0.005) and visited out-of-hours services more frequently in the preceding 9 months than the other group (0.7 vs. 0.4, p = 0.001).

**Conclusions:**

A Turkish migrant background and a low socio-economic status are associated with the fever concept "fearful". Mothers with these attributes seem to require specific and reassuring counselling as they use antipyretic drugs extensively and out-of-hours services frequently.

## Background

The subjective meaning a symptom or a disease has for a person often varies between doctors and patients. Differences between professional and common-sense knowledge as well as cultural orientations, family traditions, and socio-economic status play an important role in the way a pathophysiological process is interpreted [1,2]. Assuming that a shared understanding between patients and doctors of "what is going on" is important for an effective therapeutic relationship, these differences can have essential implications for the acceptance of a diagnosis and the treatment adherence of patients [3,4]. Hence, the way in which health professionals deal with the differences in the meaning of a symptom is an important factor in paediatric practice and for the quality of care [5].

Fever is common, especially in young children. In a study conducted in the UK, 68-74% of the interviewed parents reported episodes of high temperature at least once every 6 months in the age range of 6-56 months [6].

Furthermore, fever accounts for 20-30% of all practice visits. However, in most of these consultations nothing harmful is diagnosed [7,8].

Nevertheless, from the parents' perspective fever often causes great concern. In 1980 Schmitt introduced the term "fever phobia" to describe parents' fearful view of fever [9]. A number of studies investigated parents' knowledge, perceptions, theories, and practices of childhood fever [9-18]. A frequent finding is that parents are not correctly informed about the temperature, defining fever as a medical term [13-15,17]. They administer antipyretics incorrectly and use health care services inappropriately [10,11,14,15]. Pursell points to the benefits of increased vigilance and close attention to hydration by parents when their child has fever [18]. Several studies further showed that educational level, socio-economic status and cultural background are the main determinants of knowledge and judgement of childhood fever [11-14]. Authors reported that a higher socio-economic status and educational level, and the affiliation to the mainstream culture contributed to a more scientifically oriented knowledge of fever.

What consequences may evolve from these differences? First, parents may require different kinds of information and counselling according to their socio-economic and cultural background. This seems to be particularly relevant, since there is evidence that ethnic minority parents more often report problems in their relationship with their GP and have different beliefs about health and health care than native-born parents [19]. Second, doctors should be aware of their patients' illness concepts as it has been shown that doctors' beliefs of parental expectations can influence their treatment decisions. Several authors showed that doctors' assumed parental expectation of antibiotic treatment lead to an increased and inappropriate prescription of antibiotics [20-22].

To conclude, recognising patients as experts on their illness and taking their perspective seriously seems to be an important prerequisite for a trustful relationship and improved health outcomes [23,24].

How can research on parents' views on fever support a collaborative relationship between doctors and parents?

Most studies conducted so far compared parental views with the medical reference standard, thereby displaying the parental view as more or less deficient. Readers may solely infer from these results that parents require more information and education about the nature of fever. In many cases this is probably necessary and appropriate. However, according to the principles of patient centred medicine an understanding of the patient's illness experience is necessary to provide effective management [25-27]. Therefore this study aimed to analyse the parents' experience from their own perspective. This approach may help paediatricians to counsel parents more effectively by eliciting their explanatory models, fears and concerns.

In this study we use two different concepts of fever that were developed on the basis of qualitative interviews conducted with parents in another study prior to this one [28]. The aim of the current study was to investigate the relationship of socio-economic status and cultural background with two opposing concepts of fever, and to determine which actions are related to them. We test the hypothesis that a low socio-economic status and a Turkish background are associated with a fearful concept of fever.

## Methods

### Design

We performed a cross-sectional survey that was conducted as face-to-face interviews from February 2009 through June 2009 in an urban region of the federal state of North Rhine-Westphalia, Germany. The local ethics committee approved the study. Mothers gave written informed consent.

### Instrument

The questionnaire consisted of 36 questions and 205 items. We used two-point dichotomous scales with the scoring options yes = one, no = zero, and six-point Likert scales with the options one = fully disagree and six = fully agree. The questionnaire also contained four open-ended questions. In order to describe the socio-economic and educational status (SES) we used the instruments administered in the German health interview and examination survey for children and adolescents (KiGGS), which had been tested, validated, and allows for comparisons with our findings [29,30]. The SES consists of the following dimensions: educational level, professional position, and monthly income. Each dimension can be rated with one to seven points that are summed up to calculate the total score. The Winkler index subdivides the total score into three groups: high (15-21), medium (9-14) and low SES (3-8).

The two fever concepts "fearful" and "functional" were developed on the basis of a literature review and a qualitative interview study with 20 German and Turkish mothers (manuscript submitted). The concepts were represented in 6 statements that could be rated with a six-point Likert scale (Table 1). The criterion for the concept fearful was a total score <21 and for the concept functional >20.

**Table 1:** Items representing the fever concepts 'fearful' and 'functional'

1	Lowering temperature at the beginning prevents the development of a serious disease.
2	The body can cope with most germs itself.

3	Fever and diseases with fever are important for the healthy development of my child.

4	High fever indicates that the child's body is fighting.

5	Fever belongs to childhood - there is not much one can do.

6	The safest way to treat fever is the administration of antibiotics.

The preliminary version of the questionnaire was tested in a field pilot study (n = 50). Content validity was approved by an expert panel of paediatricians, methodologists and experts on German and Turkish culture. All but one of the items showed moderate to almost perfect agreement in test-retest reliability (Kohen's Kappa >0.4). More details about the development of the questionnaire appear elsewhere [28]. The complete questionnaires in German and Turkish are attached to this publication (additional files 1 and 2).

### Participants

In Germany children are commonly seen by paediatricians in private practices for outpatient care and not by GPs. Therefore, 35 paediatricians were invited during two quality circle meetings. Sixteen paediatrician's practices consented to participate. Additionally, we chose two outpatient clinics of regional children's hospitals as sites for the interviews. Interview sites were selected in areas of different socio-economic composition in order to capture a broad spectrum.

Mothers were approached and interviewed while waiting for a consultation with the paediatrician. They were selected as a consecutive sample on the basis of the anticipated waiting time, i.e. the mother with the longest expected waiting time was interviewed by the next available interviewer. In order to achieve a broad sample of patients a minimum of 5 participants was interviewed at each practice. For inclusion the mother had to have a child aged 6 months to 8 years. Mothers were asked to answer the questions with regard to the last fever episode of their youngest child. The reason for consultation at the day of the interview did not necessarily have to be fever. One exclusion criterion was a severely ill state of the present child, i.e. if the mother, the interviewer, or the nurse found an interview unacceptable in view of the child's state, no interview was conducted. The cultural background of the mother had to be Turkish or German. A mother was considered to have a Turkish cultural background if she or at least one of her parents was born in Turkey. A mother was considered to have a German background if she and her parents were born in Germany. Fathers or grandparents accompanying a child alone were not interviewed.

### Data collection

We conducted standardized face-to-face interviews in order to enable mothers to participate who were not proficient enough in reading and writing to fill out a questionnaire on their own. The interviews were conducted in a separate room of the practice in order to guarantee privacy after having received the mother's consent and lasted approximately 30-45 minutes. Mothers with a Turkish background were asked whether they preferred to be interviewed in German or Turkish. Therefore the questionnaire had to be translated back and forth by professional translators prior to the data collection.

All three interviewers were female. They received training before beginning and at the middle of the data collection by means of videotaped interviews with test subjects. Afterwards videos were discussed with peers and experts in interviewing.

### Statistical Analysis

Statistical analysis was performed with SPSS 18.0 for Windows. Prior to uni- and multivariate analyses a confirmatory factor analysis was applied to the six items forming the fever concepts to find out whether these items converged into a one dimensional construct as proposed by the qualitative analysis. Factor analysis was performed by means of principal components analysis and varimax rotation in order to arrive at the solution which demonstrated both the best simple structure and the most coherence. Examination of the internal consistency of the item pool was performed by calculating Cronbach's alpha coefficient for the generated factor.

Univariate analysis with Bonferroni correction was carried out to determine variables associated with concepts of childhood fever, action patterns, and concerns. The two-tailed chi-square test was used to analyze differences in proportion rates and the Mann-Whitney U test was used to analyze differences in medians among groups. The unadjusted significance level was set at α = 0.05.

Additionally, a multiple logistic regression was performed for the fever concepts using a stepwise backward selection based on the likelihood ratio statistics. Both the single fever items and the fever scale from the factor analysis were introduced as dependent variables in the logistic regression model where 20 points was defined as the splitting point. For all included variables, adjusted odds ratios (AOR) and 95% confidence intervals (CI) were calculated. Multicollinearity between the socio-economic status and the cultural background was examined using the correlation coefficient r and the tolerance factor t. The Hosmer-Lemeshow goodness-of-fit test was used to assess how well the chosen model fitted the data.

## Results

In total 369 mothers were invited to participate in the study. A total of 338 interviews were completed with 174 German and 164 Turkish mothers. The most common reason to refuse the interview was lack of interest in being interviewed (17 mothers) followed by time pressure (3 mothers).

### Demographic data

With 34.1 ± 6.3 years the average age of German mothers was significantly higher than that of Turkish mothers (32.0 ± 5.0 years; p = 0.001). The total score for the socio-economic status (SES) was 4.7 out of 21 points lower in the Turkish compared to the German group (p = 0.000) (Table 2).

**Table 2:** demographic characteristics of interviewed mothers

	German	Turkish	Total
Average age	34.1 (SD 6.3)	32.0 (SD 5.0)	33.0 (SD 5.8)

Average number of children	1.8 (SD 0.9)	2.2 (SD 1.0)	2.0 (1.0)

Socio-economic status*	13.0 (SD: 4.5)	8.3 (SD: 4.1)	10.6 (SD: 4.9)

* Median socio-economic status (Winkler index, possible values 3-21)

Among the Turkish participants 31% were born in Germany and 36.2% had German citizenship. Secondary education was completed in Germany by 52%.

### Confirmatory factor analysis for fever concepts

All 6 items were introduced to a confirmatory factor analysis which converged into one factor explaining 42.3% of the total variance. With a Kaiser-Meyer-Olkin measure of sampling adequacy of 0.778, a highly significant Bartlett test of sphericity (p < 0.001), and a Cronbach's α of 0.717, the factor can be regarded as meaningful and valid.

### Prevalence of fever concepts

#### Univariate analysis

Cultural background and socio-economic status were the only factors that showed an association with the fever concepts. Having a Turkish background and low SES contributed to the concept "fearful". Surprisingly, the number of children had no effect. Neither did the mother's age, the frequency of the child's fever periods in the preceding eight months, nor having experienced or witnessed a child's death or serious disease in the family (Table 3). Considering that cultural background is a broad concept and encompasses mothers born and raised in Germany as well as mothers immigrating to Germany as adults, we analysed the influence of the country of secondary graduation and found no effect.

**Table 3:** Sociomedical parameters in relation to the fever concepts

Mothers	Total	Functional	Fearful	P-Value
Total [N (%)]	338 (100)	227 (67,2)	111 (32,8)	

Cultural background [N (%)]				<0.001^ae^

German	174 (100)	136 (78.2)	38 (21.8)	

Turkish	164 (100)	91 (55.5)	73 (44.5)	

SES [N (%)]				0.001^ae^

Low	125 (100)	65 (52.0)	60 (48.0)	

Middle	144 (100)	107 (74.3)	37 (25.7)	

High	69 (100)	55 (79.7)	14 (20.3)	

Number of children [Median (IQR)]	2 (1; 3)	2 (1; 2)	2 (1; 3)	0.166^c^

Mother's age [average years ± SD]	33.5 ± 6.2	33.4 ± 6.0	32.3 ± 5.3	0.102^b^

Loss of child or serious disease (experience or witness) [N (%)] ^d^				0.661^a^

No	268 (100)	179 (66.8)	89 (33.2)	

Yes	69 (100)	48 (69.6)	21 (30.4)	

Frequency of fever in preceding 8 months [Median (IQR)]	2 (1; 3)	2 (1; 3)	2 (1; 4)	0.268^c^

^a ^Chi-Square test; ^b ^T test; ^c ^Mann-Whitney U test; ^d ^differing N due to missing values;

^e ^significant after Bonferroni correction

#### Multivariate analysis

Table 4 shows the adjusted odds ratios (AORs) for both the single items as well as for the fever concept scale. Multivariate analysis confirmed the results of the univariate tests. In almost all logistic regression models the AOR was significantly lower than 1 for the socio-economic status and higher than 1 for the cultural background. With values of r = -0.472 for the correlation and t = 0.763 for the tolerance between both variables there is no statistical hint for multicollinearity in our model.

**Table 4:** Determinants of fever concepts on single item level and on total score on fever scale

		Adjusted Odds-Ratios (95% CI)			
**Item**	**Item**	**Culture **^**c**^	**SES**^**b**^		**Other variables**
			
**No**.			**medium**	**high**	**Child death^d^**

1^a^	Lowering temperature	2.79 (1.76-4.41)	-	-	1.89 (1.03-3.47)

2	Body copes itself	1.95 (1.18-3.24)	0.42 (0.24-0.73)	0.35 (0.17-0.69)	^e^

3	Fever important for development	1.60 (0.96-2.67)	0.40 (0.23-0.70)	0.52 (0.26-1.02)	^e^

4	Fever means body fights	2.53 (1.40-4.59)	0.35 (0.20-0.63)	0.13 (0.05-0.36)	^e^

5	Fever normal in childhood	2.31 (1.24-4.28)	0.63 (0.34-1.15)	0.28 (0.11-0.75)	^e^

6^a^	Antibiotics safest treatment	4.37 (2.31-8.22)	0.48 (0.27-0.86)	0.19 (0.07-0.55)	^e^

Total score on fever scale (Splitting-value of 20)		1.99 (1.16-3.44)	0.53 (0.30-0.92)	0.44 (0.21-0.95)	^e^

^a^: Items were recoded in reverse order.

^b^: AORs for medium and high SES are presented in relation to low SES.

^c^: Culture represents a Turkish in relation to a German cultural background.

^d^: Child death represents having the experience of a child death in own or close family in relation to not having this experience.

^e^: No association found.

On the level of single items we found the attitude towards antipyretic drugs and antibiotics particularly interesting. Item one stated, "Lowering temperature at the beginning prevents the development of a serious disease." Item six stated that, "the safest way to treat fever is the administration of antibiotics". The first item showed a positive association with Turkish cultural background (AOR 2.79 (1.76-4.41)). Turkish mothers were also more likely to consider antibiotics to be the safest treatment in case of fever (AOR 4.37 (2.31-8.22). Furthermore the SES played an important role. Middle and high SES mothers were less likely to agree with item six (AOR 0.48 (0.27-0.86) and 0.19 (0.07-0.55)).

The fever concept total score showed the same association of fever concepts with cultural background and SES. The concept "fearful" was positively associated with a Turkish background (AOR 1.99 (1.16-3.44) and negatively with a medium and high SES (AOR 0.53 (0.30-0.92) and AOR 0.44 (0.21-0.95)).

### Actions taken by mothers and their concerns related to fever concepts

The self-reported actions taken by mothers when their child has fever showed further correlations with the concepts "fearful" and "functional". Antipyretic drugs such as acetaminophen were administered more often by mothers associated with the concept "fearful" (99.1% versus 93.4%, p = 0.02). They were also given more often before the recommended interval of 6 hours (46.8% vs. 31.3%; p = 0.005). Mothers related to the "functional" group reported a significantly lower median of 1 doctor visit in the preceding 9 months (IQR [0;3], average: 2.2) compared to a median of 2 reported visits in the group of mothers responding to the "fearful" concept (IQR [1;3], average: 2.4, p < 0.006, Mann Whitney U-Test). Interestingly, this difference is solely due to the difference in out-of-hour visits in the emergency room (average 0.7 vs. 0.4, p = 0.001 Mann Whitney U-Test). There was no significant difference in the number of visits during normal office hours (average 1.6 visits in both the fearsome and functional group).

## Discussion

The present study investigates the relationship of socio-economic status and cultural background with two fever concepts of German and Turkish mothers in an urban region of Germany. The results confirm our hypothesis that having a Turkish background and a low SES increases the likelihood of considering fever as a troublesome event. The concepts "fearful" and "functional" showed good results for reliability and internal consistency. They seem to reflect typical perspectives of mothers who are confronted with childhood fever.

The concepts are related to important aspects of patient care. Mothers leaning towards the fearful concept incline towards a higher use of antipyretic drugs with possible side effects by over dosage. On the other hand, perceiving fever as merely a normal and possibly healthy experience for children may lead to an underestimation of a child's condition by the caregivers.

In contrast to previous studies about parental fever concepts that compared parents' views with the medical reference standard, this study presents the views of parents from the parents' perspective, avoiding comparisons with normative standards by using items that have been developed on the basis of a qualitative interview study. We believe this approach could help raise awareness among health care practitioners regarding the importance of parental concepts when treating children, given these concepts' positive and sometimes negative implications for the child's health. On a more practical level the items could be used in patient care, e.g. in a pre-visit questionnaire to inform paediatricians about the parent's fever concept. This could help tailor the information given by the paediatrician to the specific views and concerns of parents. Furthermore, avoiding side effects of over dosing antipyretics, reducing costs through less out-of-hours visits, and reducing antibiotic prescriptions might be relevant consequences of effective counselling on the basis of identifying mothers' concepts of fever. However, such effects of a specific counselling strategy need to be studied in a controlled intervention study, as Cals et al. did for lower respiratory tract infections [31].

As in other studies we found the educational level and socio-economic status to play an important role in the experience of fever [9-13]. The influence of the socio-economic status on images of health in general has been described in 1984 by d'Houtaud and Field in France [32]. With regard to concepts of fever, the theory of locus of control might offer an explanation. Lachman & Weaver found that participants with low income had lower perceived mastery and more perceived constraints. Contrary to this, participants with higher income had greater mastery and a better health status [33]. Bosma confirmed these findings for paediatrics and showed that a low SES is associated with a higher prevalence of external locus of control and an absence of problem-focused coping [34]. This could explain the higher usage of out-of-hours services, as affected mothers trust less in their ability to cope with the child's disease themselves.

This study has three important limitations. First, as we compared the accounts of mothers with a non-migrant background with those of mothers with a Turkish origin there is a substantial number of other cultural backgrounds that remain unstudied. Thus, it is difficult to judge whether the differences we found are truly due to cultural differences in the sense of differences in values and shared beliefs of a group, or rather due to a difference in terms of a migration experience.

Second, the study represents the accounts participants gave of their own behaviour. Although we tried to provide a trustful environment for the interview, a bias in the sense of social desirability cannot be ruled out. Observed behaviour or prospective documentation of administered antipyretic drugs, e.g. by the mothers, would have produced more accurate results than the retrospective information mothers gave in the interview.

Third, although we aimed to recruit mothers of divergent socio-economic backgrounds, this study does not provide a representative sample statistically. Sixteen of 35 eligible paediatricians consented to participate in the study. As we did not gather data on the characteristics of non-participating practices and their reasons to refrain from participation a possible selection bias cannot be ruled out. However, comparing the distribution of socio-economic status in both groups we find the same pattern as Schenk et al did in a national representative survey conducted among 2590 subjects between 2003 and 2006 [30].

## Conclusions

This study shows that a Turkish migrant background and a low socio-economic status are associated with the fever concept "fearful". Mothers with these attributes seem to require specific and reassuring counselling as they seem to use antipyretic drugs extensively and out-of-hours services frequently. However, mothers perceiving fever as merely a normal and possibly healthy experience for children may underestimate the child's state and seek help too late, especially in infants. The items used in this study were derived from qualitative interviews with mothers and represent their experiences. Therefore, they may be applicable for counselling purposes in clinical settings.

## Competing interests

TL was supported by a grant of the Federal Ministry of Education and Research, Germany (01GK0716). There are no competing interests.

## Authors' contributions

TL was principal investigator of the study. He participated in the conception of the study, the development of the questionnaire and the data analysis. He took the lead on drafting the manuscript. MP participated in the conception of the study and the development of the questionnaire. She led the data collection. AS and BT participated in questionnaire development and data collection. ET and TO led the statistical analysis, participated in the interpretation of results and in the drafting of the manuscript. All authors read and approved the final manuscript.

## Pre-publication history

The pre-publication history for this paper can be accessed here:

http://www.biomedcentral.com/1471-2431/11/41/prepub

## Supplementary Material

Additional file 1 **Fever questionnaire in German**. The file contains the questionnaire used in face-to-face interviews with mothers who preferred to be interviewed in German. Click here for file

Additional file 2 **Fever questionnaire in Turkish**. The file contains the questionnaire used in face-to-face interviews with mothers who preferred to be interviewed in Turkish. Click here for file
