# Supplementary material for: Fearful or functional - a cross-sectional survey of the concepts of childhood fever among German and Turkish mothers in Germany
Source: BMC Pediatr. 2011 May 23;11:41. doi: 10.1186/1471-2431-11-41 (PMC3118121; doi:10.1186/1471-2431-11-41)
Supplement: Additional file 1 — Fever questionnaire in German. The file contains the questionnaire used in face-to-face interviews with mothers who preferred to be interviewed in German. [file 1471-2431-11-41-S1.DOC]

| 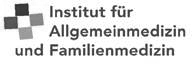 |  | 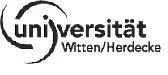 |
| --- | --- | --- |

Universität Witten/Herdecke **/** Alfred-Herrhausen-Str. 50 / 58448 Witten

**Fragebogen zum Fieber bei Kindern** (D)

Februar 2009

***INT:*** *bitte eintragen*

Datum Interviewer

Interview-Ort

| Identifier |
| --- |

**1. a) Wie Sie wissen geht es bei unserer Umfrage um Fieber beim Kind. Falls Sie**

**mehrere Kinder haben, antworten Sie bitte immer in Bezug auf Ihr jüngstes Kind.**

**Ich lese Ihnen zunächst ein paar Arten vor, die Körpertemperatur eines Kindes zu**

**bestimmen. Sagen Sie mir bitte zu jeder, ob Sie die bei Ihrem Kind anwenden**

**oder nicht.**

|  | | **ja nein** |  |
| --- | --- | --- | --- |
| 1 Ich fühle mit meiner Hand......................... | | O O 1 |  |
| 2 Ich fühle mit meiner Wange...................... | | O O 2 |  |
| 3 Ich erkenne es mit meinen Augen............ | | **O O 3** |  |
| 4 Ich messe mit einem Fieberthermometer. | | **O O 4** |  |
|  | ***INT: nur wenn Fieberthermometer*** *ausgewählt*  **b) Wenn Sie mit dem Fieberthermometer messen, wo messen Sie?** | | |
|  | Po **О**  Ohr **О** Achselhöhle **О**  Mund **О**  an einer anderen Stellen und zwar: | | |

**2. Ab welcher Temperatur sprechen Sie bei Ihrem Kind von Fieber?**

***INT:*** *genau angegeben Temperatur eintragen*

**3. Wodurch wird Fieber oder eine fieberhafte Krankheit verursacht? Ich lese Ihnen jetzt einige Möglichkeiten dazu vor. Sie sagen mir bitte zu jeder davon, ob Sie Ihrer Meinung nach als Fieberursache zutrifft oder nicht.**

| **Fieber wird verursacht durch...**  1 ... Ansteckung bei anderen Kindern oder Erwachsenen... 2 ... unzureichend warme Kleidung.......................................  3 ... Bronchitis....................................................................... 4 ... Erkältung........................................................................ 5 ... Magen-Darm-Infekt........................................................ 6 ... Blasenentzündung.........................................................  7 ... Stress im Kindergarten oder der Schule........................ 8 ... Viren............................................................................... 9 ... Bakterien........................................................................ 10 ... zu kaltes Wetter..........................................................  11 ... zu warmes Wetter........................................................  12 ... zu feuchtes Wetter.......................................................  13 ... Zahnen .........................................................................  14 ... Barfuß gehen...............................................................  15 ... Eis essen.....................................................................  16 ... Ohrenschmerzen.........................................................  17 ... familiäres Mittelmeerfieber...........................................  18 ... Impfen..........................................................................  19 ... Streit in der Familie......................................................  20 ... den Talisman zu Hause vergessen..............................  21 ... Strahlung......................................................................  22 ... den Schutzengel, der mal nicht aufgepasst hat…....... | **ja nein** O O 1 **O O 2**  **O O 3** O O 4 **O O 5**  **O O 6** O O 7 **O O 8**  **O O 9**  **O O 10**  **O O 11**  **O O 12** O O 13 **O O 14**  **O O 15** O O 16 **O O 17**  **O O 18** O O 19 **O O 20**  **O O 21**  **O O 22** |
| --- | --- |

23 Kennen Sie noch andere Fieberursachen? Wenn ja, welche?

**4.** **Welche der folgenden Maßnahmen haben Sie schon angewendet, als Ihr Kind Fieber**

**hatte?**

| 1 ... dem Kind leichte Kleidung anziehen.....................  2 ... kühle Luft zuführen................................................  3 ... Kuscheln................................................................ 4 ... Beten..................................................................... 5 ... mit seinen Eltern in einem Bett schlafen...............  6 ... das Kind warm anziehen....................................... 7 ... mit warmer Decke zudecken.................................  . 8 8 ... viel zu Trinken geben............................................ 9 ... Abwarten und Beobachten...................................  10 ... die Wünsche des Kindes erfüllen........................  11 ... in der Wohnung lassen........................................  12 ... das Kind mit kühlem Wasser baden....................  13 ... kühle Getränke zum Trinken geben....................  14 ... Kartoffeln oder Zwiebeln in die Socken geben...  15 ... kalte Umschläge z.B. Wadenwickel....................  16 ... mit Essig einreiben..............................................  17 ... mit Alkohol einreiben...........................................  18 ... einen Arzt aufsuchen...........................................  19 ... zu einem Heiler/ einer Heilerin gehen.................  20 ... zum Heilpraktiker gehen......................................  21 ... Medikamente geben............................................ | **ja nein** O O 1 **O O 2**  **O O 3** O O 4 **O O 5**  **O O 6** O O 7 **O O 8**  **O O 9**  **O O 10**  **O O 11**  **O O 12** O O 13 **O O 14**  **O O 15** O O 16 **O O 17**  **O O 18** O O 19 **O O 20**  **O O 21** |
| --- | --- |

22 Gibt es noch andere Maßnahmen die Sie bei Fieber ergreifen? Wenn ja, welche?

**5. Wenn Sie Ihrem Kind schon einmal Medikamente bei Fieber gegeben haben, welche**

**waren das?**

|  | **ja nein** |
| --- | --- |
| 1 Paracetamol oder Benuron als Zäpfchen oder Saft.... | O O 1 |
| 2 Ibuprofen, Nurofen oder Dolormin............................... | O O 2 |
| 3 Aspirin.......................................................................... | **O O 3** |
| 4 homöopathische Mittel................................................. | **O O 4** |
| 5 naturheilkundliche Mittel.............................................. | **O O 5** |
| 6 Antibiotika.................................................................... | **O O 6** |
| 7 andere Medikamente und zwar: | **O O 7** |

**6. Wenn Sie mehrfach Medikamente geben, wie viele Stunden warten Sie mindestens zwischen zwei Gaben eines fiebersenkenden Medikaments, also Paracetamol oder Ibuprofen?**

**7. Ab welcher Körpertemperatur machen Sie sich ernsthaft Sorgen wenn Ihr Kind Fieber hat?**

***INT:*** *genau angegeben Temperatur eintragen*

**8. Unter welchen Umständen machen Sie sich ernsthaft Sorgen, wenn Ihr Kind Fieber hat? Bitte sagen Sie mir zu jeder der folgenden Nennungen, ob sie zutrifft oder nicht.**

|  | **ja nein** |
| --- | --- |
| 1 Fieber sinkt trotz fiebersenkender Medikamente nicht. | O O 1 |
| 2 das Kind trinkt nicht........................................................ | O O 2 |
| 3 dem Kind geht es schlecht............................................. | **O O 3** |
| 4 das Kind hat Schüttelfrost.............................................. | **O O 4** |
| 5 das Kind schläft den ganzen Tag................................... | **O O 5** |
| 6 das Kind fantasiert......................................................... | **O O 6** |
| 7 nur wenn zum Fieber andere Krankheitsmerkmale  hinzukommen................................................................. | **O O 7** |
| 8 bei Fieber grundsätzlich immer...................................... | **O O 8** |

9 Gibt es noch andere Umstände, die Ihnen bei Fieber Sorgen bereiten? Wenn ja, welche?

**9.** **Vor welchen Folgen haben Sie sich schon einmal gefürchtet, als Ihr Kind Fieber hatte?**

|  | **ja nein** |
| --- | --- |
| 1 Fieberkrampf................................................... | O O 1 |
| 2 Gehirnschädigung........................................... | O O 2 |
| 3 Tod.................................................................. | **O O 3** |
| 4 Austrocknen.................................................... | **O O 4** |
| 5 Entwicklung einer schweren Krankheit.......... | **O O 5** |
| 6 Verwirrtheit...................................................... | **O O 6** |
| 7 Erblindung....................................................... | **O O 7** |
| 8 davor, dass das Fieber immer weiter steigt… | **O O 8** |

9 Gibt es noch andere Fieberfolgen, vor denen Sie sich schon einmal gefürchtet haben? Wenn ja, welche?

**10. *INT: Liste 1*** *vorlegen*

**Es folgen nun einige Aussagen zum Fieber.**

**Sagen Sie mir bitte zu jeder Aussage, inwieweit sie zutrifft oder nicht.**

***INT:*** *bitte Aussagen einzeln vorlesen und dann zu jeder hier die genannte Ziffer eintragen*

**trifft überhaupt nicht zu 1 2 3 4 5 6 trifft voll und ganz zu**

| 1 Gibt man gleich zu Beginn des Fiebers fiebersenkende Medikamente, wird die  Krankheit weniger schwer...................................................................................... | **1** |
| --- | --- |
| 2 Der Körper wird mit den allermeisten Krankheitserregern selbst fertig................... | **2** |
| 3 Fieber und Krankheiten mit Fieber sind wichtig für eine gesunde Entwicklung  meines Kindes......................................................................................................... | **3** |
| 4 Wenn das Kind hohes Fieber hat, bekämpft der Körper die Krankheit................... | **4** |
| 5 Mit homöopathischen Mitteln kann man den Körper unterstützen, das Fieber  zu überwinden......................................................................................................... | **5** |
| 6 Was meinem Kind am meisten hilft wenn es Fieber hat sind Nähe und  Zuwendung............................................................................................................. | **6** |
| 7 Fieber gehört zu den Krankheiten im Kindesalter dazu, daran kann man nicht  viel ändern.............................................................................................................. | **7** |
| 8 Die sicherste Art Fieber zu behandeln ist die Gabe von Antibiotika....................... | **8** |
| 9 Wenn mein Kind Fieber hat, ist es wichtig, dass es ganz viel Ruhe bekommt....... | **9** |

**11. Wie häufig hatte Ihr Kind seit Ende der letzten Sommerferien Fieber?**

mal ***INT:*** *wenn* ***kein mal*** *weiter mit* ***Frage 13***

**12. *INT: nur*** *wenn Kind* ***Fieber*** *hatte*

**a) Waren Sie dann beim Arzt, als Ihr Kind in dieser Zeit Fieber hatte?**

nein **O**

| ***INT:*** *nur wenn* ***nicht seit den Sommerferien*** *beim Kinderarzt gewesen*  **b) Wann waren Sie das letzte Mal wegen Fieber beim Kinderarzt?**  **Bitte nennen Sie mir Monat und Jahr.** |
| --- |
|  |

**ja O**

| ***INT:*** *nur wenn* ***ja***  **c) Wo waren Sie?** | **d) Wie oft?** |
| --- | --- |
| beim Kinderarzt.........................................  in der Ambulanz im Krankenhaus.............  beim kinderärztlicher Notdienst.................  woanders und zwar: | mal  mal  mal  mal |

**13. Nun habe ich noch ein paar Fragen zu Ihrem familiären Umfeld im Allgemeinen und zum Umgang der Familie mit Fieber beim Kind. Welchen Familienstand haben Sie? Sind Sie...**

|  | **ja** |
| --- | --- |
| 1 ... verheiratet.......... | O 1 |
| 2 ... ledig................... | O 2 |
| 3 ... geschieden........ | **O 3** |
| 4 ... oder verwitwet... | **O 4** |

**14. Welche der folgenden Personen leben ständig in Ihrem Haushalt? Sagen Sie mir bitte zu den Kindern auch jeweils wie alt diese sind.**

|  | **ja** | **Jahre** | **Monate** | **Wochen** |  |
| --- | --- | --- | --- | --- | --- |
| 1 Ehemann............ | O 1 | X | X | X |
| 2 Lebenspartner.... | O 2 | X | X | X |
| 3 a Tochter.............  b Tochter.............  c Tochter............. | O 3 a **O 3 b**  **O 3 c** |  |  |  |
| 5 a Sohn.................  b Sohn.................  c Sohn................. | **O 4 a**  **O 4 b**  **O 4 c** |  |  |  | ***INT bitte Eintragen:***  *Gesamtanzahl der Personen im Haushalt inkl. der befragten Mutter:* |
| 6 andere: | **O 5** |  |  |  |

**15. Haben Sie Kinder, die nicht in Ihrem Haushalt leben?**

| ***INT:*** *nur wenn* ***ja***  **b) Wie alt sind diese Kinder?** |
| --- |
|  |

nein **O** ja **O**

**16. Wo leben die Großeltern der Kinder jeweils mütterlicher und väterlicherseits? Falls**

**diese getrennt leben, antworten Sie bitte für den Teil zu dem Sie häufiger Kontakt**

**haben.**

| **mütterlicherseits**  im selben Haus.................................  bis 1 Stunde entfernt.........................  mehr als 1 Stunde entfernt................  sie leben nicht mehr / weiß nicht ...... | **ja**  **O**  **O**  **O**  **O** | **väterlicherseits**  im selben Haus..................................  bis 1 Stunde entfernt.........................  mehr als 1 Stunde entfernt................  sie leben nicht mehr / weiß nicht....... | **ja**  **O**  **O**  **O**  **O** |
| --- | --- | --- | --- |

**17. Wer geht normalerweise mit den Kindern zum Arzt? Sie oder der Vater oder gehen Sie beide gleich oft mit den Kindern zum Arzt?**

**Mit „Vater“ meinen wir den leiblichen Vater oder Personen, die an Stelle des leiblichen Vaters getreten sind, z.B. Stiefvater oder Partner.**

| Sie **O** der Vater **O** beide gleich oft **O**  eine andere Person, und zwar: |
| --- |

**18. *INT: Liste 2*** *vorlegen*

**Nun sagen Sie mir bitte wieder zu jeder der folgenden Aussagen, inwieweit sie Ihrer Ansicht nach zutreffen.**

***INT:*** *bitte Aussagen einzeln vorlesen und dann zu jeder hier die genannte Ziffer eintragen,*

*falls Personen nicht vorhanden bitte* ***X*** *eintragen*

**trifft überhaupt nicht zu 1 2 3 4 5 6 trifft voll und ganz zu**

| 1 In meiner Familie gibt es sehr unterschiedliche Vorstellungen zur Behandlung  von Fieber............................................................................................................... | **1** |
| --- | --- |
| 2 Die Großeltern passen oft auf das kranke Kind oder die anderen Kinder auf........ | **2** |
| 3 Mein Mann (oder Partner) ist oft die treibende Kraft, wenn es darum geht, mit dem fiebernden Kind zum Arzt zu fahren................................................................... | **3** |
| 4 Ratschläge aus Familie und Bekanntenkreis helfen mir nicht weiter und verunsichern mich noch mehr, wenn mein Kind Fieber hat...................................... | **4** |
| 5 Ich weiß am besten was meinem Kind bei Fieber hilft........................................... | **5** |

**19. Ist in Ihrer Familie oder in Ihrem Bekanntenkreis schon einmal ein Kind an einer schweren Krankheit erkrankt oder vielleicht sogar gestorben?**

| ***INT:*** *nur wenn* ***ja***  **b) Können Sie mir sagen an was für einer Krankheit?** |
| --- |
|  |

nein **O** ja **O**

**20. *INT: Liste 3***  *vorlegen*

**Welche drei Personen auf dieser Liste beeinflussen Sie am meisten, wenn Ihr Kind**

**Fieber hat?**

| 1 der Vater des Kindes...........  2 meine Mutter ......................  3 mein Vater ..........................  4 meine Schwiegermutter...... 5 mein Schwiegervater...........  6 meine Schwester.................  7 meine Schwägerin...............  8 andere Verwandte: | **ja** O 1O 2 **O 3**  **O 4**  **O 5**  **O 6**  **O 7**  **O 8** | 9 Freunde/innen................................  10 Kollegen/innen..............................  11 eine Ärztin / ein Arzt.....................  12 andere und zwar: | **ja**  **O 9**  **O 10**  **O 11**  **O 12** |
| --- | --- | --- | --- |

**21. *INT: Skala 1 vorlegen***

**Bitte sagen Sie mir, inwiefern die Ratschläge der eben genannten Personen Ihnen jeweils Sicherheit geben oder Sie verunsichern wenn Ihr Kind Fieber hat.**

***INT:*** *Die drei gerade ausgewählte Personen vorlesen und* *hier zu jeder Person die genannte Ziffer eintragen*

**Sicherheit 1 2 3 4 5 6 Verunsicherung**

**Ratschläge...**

| 1...vom Vater des Kindes.......  2...von der eigenen Mutter.....  3...vom eigenen Vater............  4...von der Schwiegermutter..  5...vom Schwiegervater.........  6…von meiner Schwester….  7…von meiner Schwägerin...  8...von | **1**  **2**  **3**  **4**  **5**  **6**  **7**  **8** | 9 ...von Freunden/innen  10 ...von Kollegen/innen  11...von einer Ärztin / einem Arzt  12...von | **9**  **10**  **11**  **12** |
| --- | --- | --- | --- |

**22. Zum Schluss habe ich noch ein paar Fragen zu Ihrer Person.**

**Wann sind Sie geboren? Nennen Sie mir bitte nur Monat und Jahr.**

19

**23. Welchen höchsten allgemeinbildenden Schulabschluss haben Sie?**

***INT: nur*** *falls* ***Partner*** *vorhanden:*

**... und Ihr Lebenspartner?**

***INT: jeweils*** *bitte 1 ankreuzen:*

| 1 Hauptschulabschluss/Volksschulabschluss (8. bis 10. Klasse).  2 Realschulabschluss....................................................................  3 Abschluss der Polytechnischen Oberschule (10. Klasse)..........  4 Fachhochschulreife (Abschluss einer Fachoberschule).............  5 Abitur (Gymnasium bzw. EOS; 12. oder 13. Klasse).................  6 ohne Abschluss Schule beendet................................................  7 noch keinen Schulabschluss......................................................  8 anderer Schulabschluss und zwar: | **Mutter**  **O**  **O O O O O O O** | **Partner** O 1O 2 **O 3**  **O 4**  **O 5** O 6O 7 **O 8** |
| --- | --- | --- |

**24.** **Welche der folgenden Ausbildungsabschlüsse haben Sie?**

***INT: nur*** *falls* ***Partner*** *vorhanden:*

**... und Ihr Lebenspartner?**

***INT: jeweils*** *bitte 1 ankreuzen:*

| 1 Lehre (beruflich-betriebliche Ausbildung)....................................  2 Berufsschule, Handelsschule (beruflich-schulische Ausbildung)  3 Fachschule (z.B. Meister-Technikerschule, Beruf- oder  Fachakademie)...........................................................................  4 Fachhochschule, Ingenieurschule..............................................  5 Universität, Hochschule..............................................................  6 kein beruflicher Abschluss (und auch nicht in der Ausbildung)...  7 noch in beruflicher Ausbildung (Auszubildender, Student).........  8 anderer Ausbildungsabschluss und zwar: | **Mutter**  **O**  **O**  **O O O O O O** | **Partner** O 1O 2 **O 3**  **O 4**  **O 5** O 6O 7 **O 8** |
| --- | --- | --- |

**25. Sind Sie zurzeit erwerbstätig? Unter Erwerbstätigkeit wird jede bezahlte bzw. mit einem Einkommen verbundene Tätigkeit verstanden, egal welchen zeitlichen Umfang sie hat.**

**Was trifft auf Sie zu?**

***INT: nur*** *falls* ***Partner*** *vorhanden:*

**... und was auf Ihren Lebenspartner?**

***INT: jeweils*** *bitte 1 ankreuzen:*

***INT:*** *bei* ***Voll- oder Teilzeit:***

*weiter mit* ***Frage 26***

| 1 voll erwerbstätig........................................................................................  2 teilzeitbeschäftigt ..................................................................................... | **Mutter**  **O**  **O** | **Partner** O 1O 2 |
| --- | --- | --- |

***INT:*** *nur wenn* ***einer oder beide nicht*** *Voll- oder Teilzeit erwerbstätig*

| 3 Mutterschafts-, Erziehungsurlaub, Elternzeit, Beurlaubung.....................  4 Hausfrau/ Hausmann...............................................................................  5 geringfügig erwerbstätig, Mini Job............................................................  6 Beschäftigung über die Arbeitsagentur (z.B.:„Ein-Euro-Job“)..................  7 gelegentlich oder unregelmäßig beschäftigt.............................................  8 berufliche Ausbildung/Lehre/Studium und zwar:  9 Umschulung..............................................................................................  10 Wehrdienst/Zivildienst............................................................................  11 nicht erwerbstätig (einschließlich: Arbeitslose, Vorruheständler)........... | **O**  **O O O O O O O**  **O** | **O 3**  **O 4**  **O 5** O 6O 7 **O 8**  **O 9**  **O 10**  **O 11** |
| --- | --- | --- |

**b) Waren Sie (oder war er) früher einmal voll- oder teilzeiterwerbstätig?**

ja **O** *weiter mit* ***Frage 26***

nein **O** *nur wenn* ***beide*** *nie erwerbstätig weiter mit* ***Frage 29***

**26. Welche berufliche Tätigkeit üben Sie derzeit hauptsächlich aus?**

**Wenn Sie nicht mehr erwerbstätig sind, welche Tätigkeit haben Sie bei Ihrer früheren hauptsächlichen Erwerbstätigkeit zuletzt ausgeübt?**

**Sagen Sie mir bitte jeweils für sich und Ihren Partner zu welcher Gruppe dieser Beruf**

**gehört.**

**27. Was ist Ihr Beruf?**

| 1 Selbständige Landwirt/in bzw. Genossenschaftsbauer/-bäuerin..............  2 Akademiker/in in freiem Beruf (Arzt/Ärztin, Rechtsanwalt/-anwältin,  Steuerberater/in)........................................................................................  3 Selbständig im Handel, Gewerbe, Handwerk, Industrie, Dienstleistung,  Ich-AG......................................................................................................  4 Beamter/Beamtin, Richter/in, Berufssoldat/in...........................................  5 Angestellte/r..............................................................................................  6 Arbeiter/in.................................................................................................  7 Ausbildung................................................................................................  8 Mithelfende/r Familienangehörige/r.......................................................... | **Mutter**  **O**  **O**  **O O O O O O** | **Partner** O 1 **O 2**  **O 3**  **O 4**  **O 5** O 6O 7 **O 8** |
| --- | --- | --- |

Mutter:

Vater:

**28. Was trifft auf Ihren Beruf zu?**

***INT: nur Untergruppen*** *der* *gerade* ***genannten Berufsgruppe*** *vorlesen*

| 1 Selbständige Landwirt/in bzw. Genossenschaftsbauer/-bäuerin..............  ... a mit einer landwirtschaftlich genutzten Fläche bis unter 10 ha..........  ... b mit einer landwirtschaftlich genutzten Fläche von 10 und mehr ha..  ... c Genossenschaftsbauer/-bäuerin (ehemals LPG).............................  2 Akademiker/in in freiem Beruf und habe/hatte.........................................  ... a keine weiteren Mitarbeiter/innen........................................................  ... b 1 bis 4 Mitarbeiter/innen....................................................................  ... c 5 und mehr Mitarbeiter/innen............................................................ | **Mutter**  **O**  **O**  **O O**  **O O O O** | **Partner** O 1 **O 1 a**  **O 1 b**  **O 1 c**  **O 2**  **O 2 a**  **O 2 b**  **O 2 c** |
| --- | --- | --- |

| 3 Selbständig im Handel, Gewerbe, Handwerk, Industrie, Dienstleistung,  Ich-AG und habe/hatte.............................................................................  ... a keine weiteren Mitarbeiter/innen.......................................................  ... b 1 bis 4 Mitarbeiter/innen....................................................................  ... c 5 und mehr Mitarbeiter/innen.............................................................  ... d PGH-Mitglied.....................................................................................  4 Beamter/Beamtin, Richter/in, Berufssoldat/in, und zwar .........................  ... a im einfachen Dienst (bis einschl. Oberamtsmeister/in).....................  ... b im mittleren Dienst (von Assistent/in bis einschl. Hauptsekretär/in,  Amtsinspektor/in)..................................................................................  ... c im gehobenen Dienst (von Inspektor/in bis einschl.  Oberamtsrat/-rätin).............................................................................  ... d im höheren Dienst, Richter/in (von Rat/Rätin aufwärts)....................  5 Angestellte/r, und zwar.............................................................................  ... a mit ausführender Tätigkeit nach allgemeinen Anweisungen  (z. B. Verkäufer/in, Kontorist/in, Datentypist/in)....................................  ... b mit einer qualifizierten Tätigkeit, die ich nach Anweisung erledige  (z. B. Sachbearbeiter/in, Buchhalter/in, technische/r Zeichner/in).........  ... c mit eigenständiger Leistung in verantwortlicher Tätigkeit bzw. mit  Fachverantwortung für Personal (z. B. wissenschaftliche/r  Mitarbeiter/in, Prokurist/in, Abteilungsleiter/in bzw. Meister/in im  Angestelltenverhältnis).........................................................................  ... d mit umfassenden Führungsaufgaben, Entscheidungsbefugnissen..  6 Arbeiter/in, und zwar ................................................................................  ... a ungelernt............................................................................................  ... b angelernt............................................................................................  ... c Facharbeiter/in...................................................................................  ... d Vorarbeiter/in, Kolonnenführer/in.......................................................  ... e Meister/in, Polier/in, Brigadier/in........................................................  7 Ausbildung, und zwar...............................................................................  ... a als kaufmännisch-technische/r Auszubildende/r...............................  ... b als gewerbliche/r Auszubildende/r.....................................................  ... c in sonstiger Ausbildungsrichtung.......................................................  8 Mithelfende/r Familienangehörige/r.......................................................... | **Mutter**  **O**  **O**  **O O**  **O O O**  **O**  **O**  **O**  **O**  **O**  **O**  **O O O  O**  **O**  **O**  **O**  **O**  **O O O**  **O**  **O** | **Partner** O 3 **O 3 a**  **O 3 b**  **O 3 c**  **O 3 d**  **O 4**  **O 4 a**  **O 4 b**  **O 4 c**  **O 4 d** O 5 **O 5 a**  **O 5 b**  **O 5 c**  **O 5 d**  **O 6**  **O 6 a**  **O 6 b**  **O 6 c**  **O 6 d**  **O 6 e**  **O 7**  **O 7 a**  **O 7 b**  **O 7 c**  **O 8** |
| --- | --- | --- |

**29.** ***INT: Liste 4*** *vorlegen*

**Wie hoch ist das durchschnittliche monatliche Nettoeinkommen Ihres Haushalts insgesamt? Das heißt von allen Haushaltsmitgliedern zusammen nach Abzug von Steuern und Sozialabgaben. Dazu zählen auch Erziehungsgeld, Kindergeld und Ähnliches. Bitte sagen Sie mir, welche Gruppe auf Ihr Haushaltseinkommen zutrifft.**

| unter 750 Euro.......................  750 bis unter 1 250 Euro.......  1 250 bis unter 1 750 Euro....  1 750 bis unter 2 250 Euro....  Angabe verweigert................ | **ja**  **O 1 a**  **O 1 b**  **O 2 O 3**  **O** | 2 250 bis unter 3 000 Euro.......  3 000 bis unter 4 000 Euro.......  4 000 bis unter 5 000 Euro.......  5 000 Euro und mehr...............  weiß nicht................................. | **ja**  **O 4**  **O 5**  **O 6 O 7**  **O** |
| --- | --- | --- | --- |

**30. Wie ist Ihr Kind krankenversichert?**

gesetzlich **O** privat **O** gesetzlich mit privater Zusatzversicherung **O**

anders:

**31. Wie würden Sie die Gegend beschreiben, in der Sie überwiegend aufgewachsen sind?**

ländlich **O** kleinstädtisch **O** mittelstädtisch **O** großstädtisch **O**

**32. In welchem Land ist Ihr Ehemann bzw. Ihr Partner geboren?**

Deutschland **O**

Türkei **O**

in einem anderen Land und zwar:

| ***INT: nur falls im Ausland geboren*** | |
| --- | --- |
| **b) In welchem Jahr kam er nach**  **Deutschland?** | ***INT: nur falls in der Türkei geboren***    **c) Woher kommt er in der Türkei?** |
|  | Nordtürkei **O**  Osttürkei **O**  Südtürkei **O**  Westtürkei **O**  Mittelanatolien **O** |

**33. Wie würden Sie die Gegend beschreiben, in der er aufgewachsen ist?**

ländlich **O** kleinstädtisch **O** mittelstädtisch **O** großstädtisch **O** weiß nicht **O**

**34. *INT: nur wenn Ehemann oder Partner zugewandert***

**Wenn Ihr Ehemann oder Partner aus einem anderen Land zugewandert ist, zu welcher Zuwanderungsgruppe gehört er?**

| 1 Asylbewerber............................................................................................  2 Asylberechtigter........................................................................................  3 Kriegs- oder Kontingentflüchtling..............................................................  4 Bürger eines EU-Mitgliedsstaates............................................................  5 Ehepartner oder Kind einer bereits in Deutschland lebenden Person.....  6 Gastarbeiter/Vertragsarbeiter/ausländischer Arbeitnehmer.....................  7 Deutschstämmiger (Spät-)Aussiedler.......................................................  8 Student.....................................................................................................  9 zu einer anderen Gruppe und zwar: | **ja**  **O 1**  **O 2 O 3 O 4 O 5 O 6 O 7 O 8**  **O 9** |
| --- | --- |

**35. Wie setzt sich ihr Freundeskreis zusammen?**

***INT:*** *nur* ***eine*** *Nennung möglich!*

| 1 hauptsächlich deutsch......................................  2 hauptsächlich türkisch......................................  3 zur Hälfte deutsch, zur Hälfte türkisch.............  4 verschiedene Nationalitäten.............................  5 zur Hälfte deutsch, zur Hälfte nicht-deutsch.... | **ja**  **O 1**  **O 2 O 3 O 4**  **O 5** |
| --- | --- |

**36. a) Zum Schluss möchten wir Sie fragen, ob Sie damit einverstanden wären, wenn wir uns bei Ihrem Kinderarzt über die Krankheiten Ihres Kindes erkundigen. Uns interessiert dabei zu welchen Ergebnissen er gekommen ist, wenn er Ihr Kind im letzten Jahr bei Fieber untersucht hat. Dazu benötigen wir Ihre schriftliche Einwilligung, den Namen Ihres Kinderarztes sowie den Familiennamen und das Geburtsdatum Ihres Kindes.**

**O** abgelehnt

**O** eingewilligt

| ***INT:*** *nur wenn* ***eingewilligt***  **b) Wie lauten Familien- und**  **Vorname Ihrer Kinder?** | **c) Wann sind die**  **Kinder geboren?** | **d) Wer ist der Kinderarzt**  **der Kinder?** |
| --- | --- | --- |
|  |  |  |

**- Ende -**
