# Supplementary material for: Fearful or functional - a cross-sectional survey of the concepts of childhood fever among German and Turkish mothers in Germany
Source: BMC Pediatr. 2011 May 23;11:41. doi: 10.1186/1471-2431-11-41 (PMC3118121; doi:10.1186/1471-2431-11-41)
Supplement: Additional file 2 — Fever questionnaire in Turkish. The file contains the questionnaire used in face-to-face interviews with mothers who preferred to be interviewed in Turkish. [file 1471-2431-11-41-S2.DOC]

**Almanca`dan Türkçe`ye tercüme**

**Genel Tıb ve Witten/Herdecke**

**Aile Doktorluğu Üniversitesi**

**Enstitüsü**

Witten/Herdecke Üniversitesi / Alfred-Herrhausen-Str. 50 / 58448 Witten

**Çocuklarda ateşle ilgili araştırma soruları (T)**

Şubat 2009

**Çağrı:** Lütfen doldurun

Tarih___________________ Araştırma sorularını soran___________________________

Görüşmenin yapıldığı yer__________________________________________________________

Sıfatı______________________________

1. **a) Bildiğiniz gibi anketimiz çocukta ateşle ilgilidir. Eğer birden fazla çocuğunuz varsa, lütfen daima en küçük çocuğu dikkate alarak cevap verin. Size ilkönce, çocuğun vücud sıcaklığını belirlemenin bir kaç tarzını okuyacağım. Lütfen bana her bir tarzı okuduğumda, bunu çocuğunuza tatbik edip etmediğinizi söyleyin.**

**Evet Hayır**

1 Elimle hissediyorum………………………….. **Ο Ο 1**

2 Yanağımla hissediyorum…………………….. **Ο Ο 2**

3 Gözlerimle tanıyorum………………………… **Ο Ο 3**

4 Bir termometre ile ölçüyorum………………... **Ο Ο 4**

**Çağrı: Yalnız termometre seçildiyse**

**b) Termometre ile ölçüyorsanız, nerede ölçüyorsunuz?**

Popo **Ο** Kulak **Ο** Koltukaltı **Ο** Ağız **Ο**

Başka bir yerde ise neresi:_____________________________

1. **Hangi vucut sıcaklığından itibaren çocuğunuzun ateşi olduğundan bahsediyorsunuz?**

**Çağrı:** Kesin olarak bildirilen sıcaklığı yazın.

___________________________________

1. **Neler ateşe veya ateşli bir hastalığa sebeb olur? Şimdi size bununla ilgili bir kaç ihtimal sayacağım. Siz bana lütfen bunlardan hangilerinin sizce ateşin sebebi olup olmadığını söyleyin.**

**Ateşe sebep olur………. Evet Hayır**

1…Diğer çocuklardan veya yetişkinlerden hastalık bulaşması……. **Ο Ο 1**

2…Yeterince sıcak olmayan kıyafet………………………………… **Ο Ο 2**

3…Bronşit……………………………………………………………. **Ο Ο 3**

4…Soğuk algınlığı…………………………………………………… **Ο Ο 4**

5…Mide-bağırsak-enfeksiyonu………………………………………. **Ο Ο 5**

6…İdrar torbası iltihabı……………………………………………... **Ο Ο 6**

7…Ana okulunda veya okulda stres……………………………….. **Ο Ο 7**

8…Virüsler…………………………………………………………… **Ο Ο 8**

9…Bakteriler…………………………………………………………. **Ο Ο 9**

10..Çok soğuk hava…………………………………………………... **Ο Ο 10**

11..Çok sıcak hava…………………………………………………… **Ο Ο 11**

12..Çok rutubetli hava……………………………………………….. **Ο Ο 12**

13. Diş çıkartma……………………………………………………... **Ο Ο 13**

14..Yalınayak gezmek……………………………………………….. . **Ο Ο 14**

15..Dondurma yemek………………………………………………. **Ο Ο 15**

16..Kulak ağrıları……………………………………………………. **Ο Ο 16**

17..Ailevi akdeniz ateşi………………………………………........... **Ο Ο 17**

18..Aşılar…………………………………………………………….. **Ο Ο 18**

19..Aile içindeki kavgalar………………………………………….. **Ο Ο 19**

20. Tılsımını evde unutmak………………………………………… **Ο Ο 20**

21..Işınlar…………………………………………………………….. **Ο Ο 21**

22.. Koruyucu meleğin bazen dikkat etmemesi……………………… **Ο Ο 22**

22a. Maşallah (Allah korusun) demeyi unutmak…………………... **Ο Ο 22a**

22b.Nazar (kötü, kem gözler)……………………………………….. **Ο Ο 22b**

23..Ateşe sebeb olan başka bir şey biliyor musunuz? Evet ise, hangisi?

_________________________________________________________________________

1. **Çocuğunuzun ateşi olduğunda, aşağıdaki tedbirlerden hangilerini aldınız?**

**Evet Hayır**

1…Çocuğa hafif kıyafet giydirmek……………………………….. **Ο Ο 1**

2…Serin hava aldırmak…………………………………………….. **Ο Ο 2**

3…Çocuğa ilgi göstermek, kucaklamak…………………………….. **Ο Ο 3**

4…Dua etmek……..………………………………………………… **Ο Ο 4**

5…Anne-babayla bir yatakta yatmak……………………………… **Ο Ο 5**

6…Çocuğa sıcak kıyafet giydirmek………………………………... **Ο Ο 6**

7…Sıcak bir örtüyle üstünü örtmek………………………………. **Ο Ο 7**

8…Çok içecek vermek……………………………………………... **Ο Ο 8**

9…Beklemek ve gözetim altında tutmak……………..…………… **Ο Ο 9**

10..Çocuğun isteklerini yerine getirmek………………………….... **Ο Ο 10**

11..Evde bırakmak…………………………………………………… **Ο Ο 11**

12..Çocuğu serin suyla yıkamak……………………………………. **Ο Ο 12**

13..Serinletici içecekler vermek…………………………………… . **Ο Ο 13**

14..Çorabının içine patates veya soğan koymak………….………. **Ο Ο 14**

15.. Soğuk şeyler sarmak, mesela baldırına……………………… **Ο Ο 15**

16..Sirkeyle ovmak…………………………………………………. **Ο Ο 16**

17.. Alkol ile ovmak………………………………………………… **Ο Ο 17**

17a.Nazar boncuğu (mavi göz) takmak……………………………. **Ο Ο 17a**

17b.Muska takmak………………………………………………….. **Ο Ο 17b**

17c.Kur`an okumak (okuyup üflemek)……………………………… **Ο Ο 17c**

18..Doktora götürmek……………………………………………….. **Ο Ο 18**

19..Hocaya götürmek…………………………………..…………….. **Ο Ο 19**

20..Şifalı bitkiler uzmanına götürmek……………………………….. **Ο Ο 20**

21.. ilaçlar vermek………………………………………..…………… **Ο Ο 21**

22..Ateşli olunca, aldığınız yaptığınız başka uygulamalar var mı? Varsa, hangileri?

1. **Daha önceden çocuğunuza ateşi olduğunda ilaçlar verdiyseniz, bunlar hangileriydi?**

**Evet Hayır**

1 Paracetamol veya Benuron şurub veya fitil olarak ……… **Ο Ο 1**

2 Ibuprofen, Nurofen veya Dolormin……………………….. **Ο Ο 2**

3 Aspirin……………………………………………………….. **Ο Ο 3**

4 Homöopatik maddeler……………………………..…… **Ο Ο 4**

5 Yabani otlardan yapılan bitkisel ilaçlar……….…… **Ο Ο 5**

6 Antibiyotik……………………………………………… **Ο Ο 6**

7 Başka ilaçlarsa, hangileri?_______________________ **Ο Ο 7**

1. **Eğer birden fazla ateş düşürücü ilaç veriyorsanız, iki veriliş arasında (mesela paracetamol veya ibuprofen) en azından kaç saat bekliyorsunuz?**

______________________________________

1. **Eğer çocuğunuzun ateşi varsa, hangi vücud sıcaklığından itibaren ciddi olarak endişeleniyorsunuz?**

**Çağrı:** Kesin olarak verilen sıcaklığı yazın

____________________________________________________

1. **Çocuğunuzun ateşi olduğunda, hangi şartlar altında ciddi olarak endişeleniyorsunuz? Aşağıda sayılanların her birisinin doğru olup olmadığını lütfen söyleyin.**

**Evet Hayır**

1 Ateş, ateş düşürücü ilaçlara rağmen düşmüyorsa…. **Ο Ο 1**

2 Çocuk bir şey içmiyorsa……………………………... **Ο Ο 2**

3 Çocuğun rahatsız ise……………..………………… **Ο Ο 3**

4 Çocuğu sıtma tutmuş ise…………..………………… **Ο Ο 4**

5 Çocuk bütün gün uyuyorsa…………………………. **Ο Ο 5**

6 Çocuk hayaller görüyorsa…………………………... **Ο Ο 6**

7 Ateşin yanı sıra başka hastalık belirtileri varsa……….… **Ο Ο 7**

8 Ateşi olunca, esas olarak daima ………………………... **Ο Ο 8**

9 Ateş olduğunda sizi endişeye sevkeden başka şartlar var mı? Varsa, hangileri?

1. **Çocuğunuzun ateşi olduğunda, hangi sonuçların ortaya çıkmasından korktunuz?**

**Evet Hayır**

1 Havale geçirmesi…………………………………………. **Ο Ο 1**

2 Beynin zarar görmesi………………………………….. **Ο Ο 2**

3 Ölüm……………………………………………………. **Ο Ο 3**

4 Kuruyup gitmek………………………………………... **Ο Ο 4**

5 Ağır bir hastalığın gelişmesi…………….…………….. **Ο Ο 5**

6 Sersemlemek……..……………………………………... **Ο Ο 6**

7 Kör olmak………………………………………………. **Ο Ο 7**

8 Ateşin git gide yükselmesi……………………………... **Ο Ο 8**

9 Ateş sonucu ortaya çıkmasından korktuğunuz başka sonuçlar var mı? Varsa, hangisi?__________________________________________________________

1. **Çağrı:** Liste 1` i önüne koyun

**Şimdi ateşle ilgili bir kaç ifade sayacağım. Lütfen bu ifadelerden her birinin ne derece doğru olup olmadığını söyleyin.**

**Çağrı:** Lütfen ifadeleri tek tek okuyun ve ondan sonra burada verilen rakamlardan birini yazın.

**Hiç doğru değil 1 2 3 4 5 6 Tamamen doğru**

| 1 Hemen ateş başlangıcında ateş düşürücü ilaçlar verilirse, hastalık daha  az ağır olur**…………………………………………………………………**  2 Vücud hastalık mikroplarının çoğu ile kendisi başeder…………………  3 Ateş ve ateşli hastalıklar, çocuğumun sağlıklı gelişimi için önemlidir…  4 Çocuğun yüksek ateşi varsa, vücud hastalıkla mücadele ediyordur……..  5 Homöopatik maddelerle, vücudun ateşi yenmesi desteklenebilir………...  6 Ateşi olunca, çocuğuma en çok yardımcı olan şeyler, ona gösterilen yakınlık  ve ilgidir……………………………………………………………………..  7 Ateş çocukluk hastalıklarının pek değiştirilemeyecek bir parçasıdır………..  8 Ateşi tedavi etmenin en emin yolu, antibiyotik vermektir………...  9 Çocuğumun ateşi olduğunda, çokça dinlenmesi önemlidir…………………………………………………………………… | **_________1**  ________ _**2**  _________ **3**  __________**4**  __________**5**  _________**6**  __________**7**  __________**8**  __________**9** |
| --- | --- |

1. **En son yaz tatilinin sonundan beri çocuğunuzun kaç defa ateşi oldu?**

__________defa **Çağrı:** Hiç ateşi olmadıysa, soru 13` e geçin

| 1. 12. **Çağrı**: Sadece çocuğun ateşi olduysa 2. **Bu zaman içinde ateşi olduğunda doktora gittiniz mi?** |
| --- |

Hayır **Ο**

| **Çağrı**: Sadece yaz tatilinden beri doktora gitmediyse   1. **Son defa ne zaman ateşten dolayı çocuk doktoruna gittiniz? Lütfen ay ve yıl olarak söyleyin.** |
| --- |
|  |

**Evet** Ο

| **Çağrı:** Sadece evet ise   1. **Nereye gittiniz?** | 1. **Kaç defa?** |
| --- | --- |
| Çocuk doktoruna……………………………..  Hastahanenin acil servisine………..  Çocuk doktoru acil servisine………………...  Başka bir yer ise, nereye__________________ | ______________defa  ______________defa  ______________defa  ______________defa |

**13. Şimdi genel olarak aile çevreniz ve ailenin ateşe karşı nasıl davdandığı ile ilgili bir kaç sorum daha olacak. Medeni haliniz nedir? Siz**

**Evet**

1…Evli misiniz?………… **Ο 1**

2…Bekar mısınız?………. **Ο 2**

3…Boşandınız mı?........... **Ο 3**

4…veya dul musunuz?.... **Ο 4**

1. **Aşağıdaki kişilerden hangileri devamlı olarak sizinle aynı evde yaşıyor? Lütfen çocukların kaç yaşında olduklarını da söyleyin.**

|  | **Evet** | **Yıl** | **Ay** | **Hafta** |  |
| --- | --- | --- | --- | --- | --- |
| 1 Evli kocam….................... | **Ο 1** | **X** | **X** | **X** |  |
| .2 Hayat arkadaşım………... | **Ο 2** | **X** | **X** | **X** |
| 3 a Kızım………………….  b Kızım………………….  c Kızım…………………. | **Ο 3a**  **Ο 3b**  **Ο 3c** |  |  |  |
| 4 a Oğlum…………………  b Oğlum…………………  c Oğlum…………………  5 Diğerleri______________ | **Ο 4a**  **Ο 4b**  **Ο 4c**  **Ο 5** |  |  |  | **Çağrı**: Lütfen doldurun  Soruları cevaplayan anne  dahil evdeki toplam nüfus _______________ |

1. **Evinizde yaşamayan çocuğunuz var mı?**

Hayır **Ο**  Evet **Ο** **Çağrı:** Sadece **evet** ise

**b)** **Bu çocuklar kaç yaşında dır?**

_________________________________________

1. **Çocuğun nine ve dedesi nerede yaşıyorlar ? Eğer ayrı yaşıyorlarsa, daha sık ilişkiniz olan tarafı dikkate alarak cevap veriniz.**

**Anne Tarafı Evet Baba Tarafı Evet**

Aynı evde……………………….. **Ο** Aynı evde……………………… **Ο**

1 Saate kadar uzaklıkta……....... **Ο**  1 Saate kadar uzaklıkta………. **Ο**

1 Saatten fazla uzaklıkta………. **Ο**  1 Saatten fazla uzaklıkta……… **Ο**

Artık hayatta değiller/Bilmiyorum **Ο** Artık hayatta değiller/Bilmiyorum **Ο**

| 1. **Normal olarak çocuklarla doktora kim gidiyor? Siz mi veya baba mı, yoksa her ikinizde aynı sıklıkta çocuklarla doktora gidiyor musunuz?**   **“Baba” ile asıl babayı veya asıl baba yerine geçmiş olan örneğin üvey baba veya hayat arkadaşınızı kastediyoruz.** |
| --- |

Siz **Ο** Baba **Ο** Her ikimiz de aynı oranda **Ο**

Başka birisiyse, kim?_______________________________________

| 1. **Çağrı:** **Liste 2**` yi önüne koyun.   **Lütfen bana aşağıdaki ifadelerden hangisinin, sizin görüşünüze göre, ne derece doğru (isabetli) olduğunu söyleyin.**  **Çağrı:** Lütfen ifadeleri tek tek okuyun ve ondan sonra, adı geçen rakamı yazın. Eğer şahıslar yoksa, X yazın. |
| --- |

**Hiç doğru değil 1 2 3 4 5 6 Tamamen doğru**

| 1. 1 Ailemde ateşin tedavisiyle ilgili çok değişik tasavvurlar (düşünceler)   Var…………………………………………………………………………. | ________**1** |
| --- | --- |
| 1. nine, dedesi sıkça hasta çocuğa veya  çocuklara bakarlar. | ________**2** |
| 1. Eşim veya hayat arkadaşım ateşi olan çocuğu doktora götürme konusunda,   genellikle sürükleyici güçtür…………………………………………………… | ________**3** |
| 1. Çocuğumun yüksek ateşi varsa, ailemden veya tanıdık çevremden verilen   Öğütler, bana yardımcı olmuyor ve bana daha da güvensizlik veriyor…………………………………………………………………………. | _________**4** |
| 5 Çocuğumun ateşi olunca, ona neyin iyi geleceğini en iyi ben biliyorum…………... | _________**5** |
| 6 Doktora giderken, bana tercümanlık yapacak birini yanımda götürüyorum…... ________6 | _________**6** |
|  |  |
| 1. **Ailenizde veya tanıdık çevrenizde daha önce hiç ağır   bir hastalığa yakalanan veya belki hatta ölen bir çocuk oldu mu?** |  |

Hayır **Ο** Evet **Ο** **Çağrı:** Sadece evet ise

| b) **Nasıl bir hastalık olduğunu bana söyleyebilir misiniz?** | |
| --- | --- |
|  |  |

| 1. **Çağrı:** Liste 3 `ü önüne koyun.   **Çocuğunuzun ateşi olduğunda, listede ki hangi üç kişi sizi en çok etkiliyor?** |
| --- |

**Evet Evet**

1 Çocuğun babası………… **Ο 1** 9 Arkadaşlar……………………….. **Ο 9**

2 Annem………………….. **Ο 2** 10 Meslektaşlarım………………….. **Ο 10**

3 Babam………………….. **Ο 3** 11 Bir doktor (hekim)……………... **Ο 11**

4 Kayınanam……………... **Ο 4** 12 Başka biri ise, kim? ______________**Ο 12**

5 Kayınbabam…………… **Ο 5**

6 Kızkardeşim…………… **Ο 6**

7 Görümcem/Eltim……….. **Ο 7**

8 Diğer akrabalar………… **Ο 8**

| 1. **Çağrı:** Skala 1`i önüne koyun.   **Çocuğunuzun yüksek ateşi olduğunda, biraz önce saydığınız kişilerin öğütleri, size ne kadar güven veya güvensizlik veriyor? Bunu lütfen bana söyleyin**.  **Çağrı:** Biraz önce seçilen üç kişiyi okuyun ve her şahsın yanına söylenen rakamı yazın. |
| --- |

**Güven veriyor. 1 2 3 4 5 6 Gevensizlik veriyor**

**Öğütleri….**

1..Çocuğun babasının…… _______**1**  9..Arkadaşlarımın…………….. _______**9**

2.. Kendi annemin……….. _______**2** 10..Meslektaşlarımın………….. _______**10**

3..Kendi babamın………. _______**3**  11..Bir doktorun (hekimin)…… _______**11**

4..Kayınanamın…………. _______**4** 12..Başka birinin ise, kimin?..... ________**12**

5..Kayınbabamın………... ________**5**

6..Kız kardeşimin……… _______ **6**

7..Görümcemin/Eltimin… _______**7**

8..Diğer akrabalarımın…. ________**8**  Kimin?___________________

| 1. **Son olarak sizin şahsınızla ilgili bir kaç sorum var.**   **Doğum tarihiniz nedir? Lütfen sadece ay ve yılı söyleyin.** |
| --- |

___________19_____

| 1. **Genel eğitim veren okulların hangisinden mezun oldunuz (hangisini bitirdiniz)?**   **Çağrı:** Sadece eşi varsa.  **… ve eşiniz hangisinden mezun?**  **Çağrı:** Yalnız bir cevap işaretleyin |
| --- |

**Türkiye`de: Anne Eşi**

1a 8. Sınıf dahil ilk öğretim **Ο Ο 1a**

1b 3 yıllık lise **Ο Ο 1b**

1c 3 yıllık teknik-meslek lisesi **Ο Ο 1c**

1d 4 yıllık teknik lise (sanat okulu) **Ο Ο 1d**

**Almanya`da:**

1 8. ile 10. Sınıf arası dahil Temel Eğitim Okulu (Hauptschule)………… **Ο Ο 1**

2 Orta okul (Realschule)…………………………………………………….. **Ο Ο 2**

3 Çok yönlü Teknik okulunun 10. Sınıfından (Polytechnische Oberschule).. **Ο Ο 3**

4 Meslek Teknik Okulu (Fachoberschule, Fachhochschulreife)…………….. **Ο Ο 4**

5 Lise (12. Veya 13. Sınıftan) (Abitur, Allgemeine Hochschulreife)……… **Ο Ο 5**

6 Bitirmeden okulu bıraktım………………………………………………….**Ο Ο 6**

7 Henüz hiç bir okulu bitirmedim……………………………………………..**Ο Ο 7**

8 Başka bir okul ise, hangisi?_____________________________________ **Ο Ο 8**

| 1. **Aşağıdaki meslek (mezuniyet= bitirme) derecelerinden hangisine sahibsiniz?**   **Çağrı:** Sadece eşi varsa.  **…..ve eşiniz hangisine sahip?**  **Çağrı:** Yalnız bir cevap işaretleyin. |
| --- |

**Anne Eşi**

1 Çıraklık eğitimi (İşletme- meslek eğitimi)……………………………………**Ο Ο 1**

2 Meslek okulu, Ticaret Meslek Lisesi……………………….………………....**Ο Ο 2**

3 Meslek akademisi, Usta-Teknisyen okulu…………………………………….**Ο Ο 3**

4 Yüksek okul , lisans eğitimi ………………………………………………….**Ο Ο 4**

5 Üniversite, yüksek okul……………………….………………………………**Ο Ο 5**

6 Hiç bir meslek mezuniyetim yok……………………………………………...**Ο Ο 6**

7 Henüz meslek eğitimi yapıyorum (öğrenci, çırak)…………………………...**Ο Ο 7**

8 Başka bir okulu bitirdiyseniz, hangisi?______________________________ **Ο Ο 8**

| 1. **Şu anda bir işte çalışıyor musunuz? Süresi ne kadar olursa olsun, parası ödenen veyahutta gelir getiren her faaliyet iş`tir. Sizin için aşağıdakilerden hangisi doğru?**   **Çağrı:** Sadece eşi varsa  **…ve eşiniz için hangisi doğru?**  **Çağrı:** Lütfen herbiri için bir cevap işaretleyin. |
| --- |

**Anne Eşi**

1 Tam gün çalışma………………………………………………………… **Ο Ο 1**

2 Yarim gün çalışma……………………………………………………………… **Ο Ο 2**

| **Çağrı:** Tam kadro veya kısmi çalışmaysa, **soru 26`ya** geçin. |
| --- |

3 Annelik, çocuk eğitme izni, parasız izin…………………………………. **Ο Ο 3**

4 Ev kadını / erkeği…………………………………………………………. **Ο Ο 4**

5 Küçük işlerde çalışma (Mini Job)……………………………………….. **Ο Ο 5**

6 İş ve İşçi Bulma Kurumu üzerinden çalışma (Ein-Euro-Job)…………….. **Ο Ο 6**

7 Arada sırada veya düzensiz olarak çalışma…………………………….. **Ο Ο 7**

8 Meslek eğitimi/ çıraklık/ öğrenci ise ne?__________________________ **Ο Ο 8**

9 Meslek değiştirmek için yeni meslek eğitimi……………………………... **Ο Ο 9**

10 Askerlik veya sivil hizmet……………………………………………….. **Ο Ο 10**

11 Çalışmama (işsizlik ve erken emeklilik dahil)………………………….. **Ο Ο 11**

| **Çağrı:** Sadece birisi veya her ikisi de tam kadro veya kısmi çalışmıyorsa  **b) Siz veya eşiniz daha önce hiç tam gün veya kısmi olarak çalıştınız mı?** |
| --- |

Evet **Ο** **Soru 26`** ya geçin

Hayır **Ο** Sadece **her ikisi** de hiç çalışmadıysa **soru 29**`a geçin

| 1. **Şu anda esas olarak nasıl bir işte çalışıyorsunuz? Artık çalışmıyorsanız, daha önceki işinizde esas olarak ne yaptınız?**   **Bana lütfen kendiniz ve eşiniz için bu mesleğin hangi gruba dahil olduğunu söyleyin.** |
| --- |

**Anne Eşi**

1 Serbest çiftçi veyahut kooperatif üyesi çiftçi……………………………….. **Ο Ο 1**

2 Serbest çalışan akademisyen (doktor, avukat veya vergi danışmanı)………. **Ο Ο 2**

3 Serbest ticaret, sanat/zanaat, sanayi, hizmet veya Ben-AŞ (Ich-AG)………. **Ο Ο 3**

4 Devlet memuru, hakim, meslekten asker……………………………………. **Ο Ο 4**

5 Sözleşmeli memur……………………………………………………………. **Ο Ο 5**

6 İşçi……………………………………………………………………………. **Ο Ο 6**

7 Meslek eğitimi………………………………………………………………. **Ο Ο 7**

8 İşte yardımcı olan aile mensubu……………………………………………. **Ο Ο 8**

1. **Mesleğiniz nedir?**

Anne: ______________________________

Eşi : ______________________________

| 1. **Mesleğinizle ilgi aşağıdakilerden hangisi doğru?**   **Çağrı:** Sadece biraz önce **söylenen meslek grubunun alt gruplarını** okuyun |
| --- |

**Anne Eşi**

1 Serbest çiftçi veyahut kooperatif üyesi çiftçi………………………………. **Ο Ο 1**

…a 10 hektardan daha küçük bir tarım arazisinde………………………… **Ο Ο 1a**

…b 10 hektar ve daha büyük bir tarım arazisinde…………………………. **Ο Ο 1b**

…c Kooperatif üyesi çiftçi (eskiden LPG)……………………………… **Ο Ο 1c**

2 Serbest çalışan akademisyen ve yanında aşağıdaki kişileri çalıştırıyordu… **Ο Ο 2**

…a Yanında başka çalışan yoktu…………………………………………… **Ο Ο 2a**

…b 1 ila 4 kişi………………………………………………………………. **Ο Ο 2b**

…c 5 ve daha fazla kişi……………………………………………………... **Ο Ο 2c**

**Anne Eşi**

3 Serbest ticaret, sanat/zanaat, sanayi, hizmet ve yanında çalışan……………. **Ο Ο 3**

…a hiç kimse …………………………………………………………. **Ο Ο 3a**

…b 1 ila 4 Çalışan……………………………………………………… **Ο Ο 3b**

…c 5 ve daha fazla Çalışan ……………………………………………….. **Ο Ο 3c**

…d PHG-üyesi……………………………………………………………… **Ο Ο 3d**

4 Devlet memuru, hakim, meslekten asker…………………………………… **Ο Ο 4**

…a En alt kademede…………………………………………………………. **Ο Ο 4a**

…b Orta kademede (asistan, kalem müdürü, müfettiş)…………………… **Ο Ο 4b**

…c Üst kademede (müfettiş, müsteşar)……………………………………. **Ο Ο 4c**

…d Daha üst kademede , hakim (müsteşar ve üstü)………………………... **Ο Ο 4d**

5 Sözleşmeli memur…………………………………………………………… **Ο Ο 5**

…a Genel talimatlara göre uygulayıcı bir görevde (örnek: Satıcı, kasadar,

işlemci ………………………….. **Ο Ο 5a**

…b Talimata göre yaptığım kalifiye bir iş

(Örnek: Muhasebeci, teknik resimci v.s. gibi)…………………………… **Ο Ο 5b**

…c Sorumlu veyahut personel sorumluluğu olan bağımsız bir görev

(Örnek: Bilimsel araştırmacı, vekil, kısım başkanı, sözleşmeli usta)……... **Ο Ο 5c**

…d Geniş yönetim ve karar verme yetkili……………………………………. **Ο Ο 5d**

6 İşçi……………………………………………………………………………. **Ο Ο 6**

…a Vasıfsıs işçi……………………………………………………………….. **Ο Ο 6a**

…b İşini yaparak öğrenmiş……………………………………………………. **Ο Ο 6b**

…c Usta işçi…………………………………………………………………... **Ο Ο 6c**

…d Ekib başı usta…………………………………………………………….. **Ο Ο 6d**

…e Ustabaşı…………………………………………………………….. **Ο Ο 6e**

7 Meslek eğitimi………………………………………………………………… **Ο Ο 7**

…a Ticari-teknik meslek eğitimi……………………………………………… **Ο Ο 7a**

…b Zanaat meslek eğitimi…………………………………………………….. **Ο Ο 7b**

…c Diğer bir meslek eğitimi………………………………………………….. **Ο Ο 7c**

8 İşte yardımcı olan aile mensubu……………………………………………… **Ο Ο 8**

| 1. **Çağrı:** Liste 4`ü önüne koyun   **Evinizin ortalama toplam aylık net geliri ne kadardır? Yani vergi ve sosyal güvenlik kesintileri düşüldükten sonra bütün (aynı evde oturan) Ev mensublarının gelirinin toplamı. Buna çocuk parası, çocuk eğitim ödeneği benzeri ödemeler de dahildir. Lütfen aşağıdakilerden hangi**  **grubun sizin evinizin geliri için doğru olduğunu söyleyin.** |
| --- |

**Evet Evet**

750 Euro`nun altında…………… **Ο 1a** 2250 ile 3000 Euro arası…………. **Ο 4**

750 ile 1250 Euro arası………… **Ο 1b** 3000 ile 4000 Euro arası………... **Ο 5**

1250 ile 1750 Euro arası………. **Ο 2** 4000 ile 5000 Euro arası………… **Ο 6**

1750 ile 2250 Euro arası……… **Ο 3** 5000 Euro ve üzeri………………. **Ο 7**

Bilgi vermedi…………………… **Ο** Bilmiyor……………………………. **Ο**

1. **Çocuğunuzun nasıl bir sağlık sigortası var?**

Yasal **Ο** Özel **Ο** Özel ek sigortalı yasal **Ο**

Başka:_______________________________________

30.**a) Siz ve eşiniz hangi ülkenin vatandaşlığına sahipsiniz?**

**Anne Eşi**

1 Türk………………………… **Ο Ο 1**

2 Alman……………………… **Ο Ο 2**

3 Çifte vatandaş……………… **Ο Ο 3**

4 Hiç bir tabiyetim yok……… **Ο Ο 4**

5 Başka ise, ne? **Ο 5**

_____________________

30.**b) Ana diliniz hangi dildir?**

Türkçe **Ο** Almanca **Ο** Kürtçe **Ο** Başka:___________________

30 **c) Hangi ülkede doğdunuz?**

**Ο** Almanya

**Ο** Türkiye **Çağrı:** Sadece “**Türkiye`de doğdum**” seçildiyse

| **b) Hangi yılda Almanya`ya geldiniz?** | **c) Türkiye`nin neresinden geliyorsunuz?** |
| --- | --- |
|  | Kuzey Türkiye **Ο**  Doğu Türkiye **Ο**  Güney Türkiye **Ο**  Batı Türkiye **Ο**  Orta Türkiye **Ο** |

1. **Çoğunlukla yetiştiğiniz çevreyi nasıl vasıflandırırsınız (tasvir edersiniz) ?**

Kırsal kesim **Ο**  Kasaba **Ο** şehir **Ο**  Büyük şehir **Ο**

31.**a) Babanız hangi ülkede doğmuş?**

Türkiye **Ο** Almanya **Ο**  Başka bir ülke:________________________

31.**b) Anneniz hangi ülkede doğmuş?**

Türkiye **Ο** Almanya **Ο**  Başka bir ülke:________________________

1. **Kocanız yahut eşiniz hangi ülkede doğmuş?**

**Ο** Almanya

**Ο** Türkiye

Başka bir ülkede ise, hangisi?_____________________________

**Çağrı: Sadece Almanya dışında doğduysa**

| **b) Hangi yılda Almanya`ya gelmiş?** | **Çağrı: sadece Türkiye`de doğduysa**  **c) Türkiye`nin neresinden geliyor?** |
| --- | --- |
|  | Kuzey Türkiye **Ο**  Doğu Türkiye **Ο**  Güney Türkiye **Ο**  Batı Türkiye **Ο**  Orta Türkiye **Ο** |

1. **Onun çoğunlukla büyüdüğü çevreyi nasıl vasıflandırırsınız (tasvir edersiniz)?**

**Kırsal kesim Ο Kasaba Ο şehir Ο Büyük şehir Ο Bilmiyorum Ο**

| 1. **Çağrı: Sadece soruları cevaplandıran veya eşi sonradan Almanya`ya geldiyse**   **Eğer siz ve/veya eşiniz başka bir ülkeden Almanya`ya geldiyseniz, hangi göçmen grubuna dahilsiniz?** |
| --- |

**Anne Eşi**

1 Sığınma (iltica) talebinde bulunanlar……………………………………………… **Ο Ο 1**

2 Sığınma hakkına sahip olanlar (mülteci= sığınmacı)……………………………… **Ο Ο 2**

3 Savaştan kaçanlar veya kontenjan dolayısıyla kabul edilmiş kaçkınlar…………. **Ο Ο 3**

4 AB (Avrupa Birliği) üyesi bir ülke vatandaşları………………………………….. **Ο Ο 4**

5 Daha önceden Almanya`da yaşayan bir şahsın çocuğu veya eşi………………….. **Ο Ο 5**

6 Misafir işçi / Sözleşmeli işçi / Yabancı işçi………………………………………. **Ο Ο 6**

7 Alman asıllı göçmenler……………………………………………………………. **Ο Ο 7**

8 Öğrenci……………………………………………………………………………. **Ο Ο 8**

9 Başka bir gruptansa, hangisi?_________________________________________ **Ο Ο 9**

**34.a) Kocanızla / Eşinizle en çok hangi dili konuşuyorsunuz?**

Türkçe **Ο**  Almanca **Ο** Kürtçe **Ο**  Başka bir dil:____________________

**34.b) Çocuklarınızla en çok hangi dili konuşuyorsunuz?**

Türkçe **Ο**  Almanca **Ο** Kürtçe **Ο**  Başka bir dil:____________________

| 1. **Arkadaş çevreniz kimlerden oluşuyor?**   **Çağrı:** Sadece bir cevap işaretleyin |
| --- |

**Evet**

1 Çoğunlukla Almanlardan…………………………………..………… **Ο 1**

2 Çoğunlukla Türklerden…………………………………………….. **Ο 2**

3 Yarısı Alman, yarısı Türklerden………………………………….. **Ο 3**

4 Çeşitli milliyetlerden……………………………………………... **Ο 4**

5 Yarısı Alman, yarısı Alman olmayanlardan…………………….. **Ο 5**

| **35.a) Çağrı:** Liste 5`i önüne koyun.  **Lütfen bana, aşağıdaki ifadeleri ne kadar doğru bulduğunuzu söyleyin** |
| --- |

**Hiç doğru bulmuyorum 1 2 3 4 5 6 Tamamen doğru buluyorum**

1. Türkiye`de kendimi en rahat hissediyorum…………………..…………… ___________**1**
2. Daha sonra Türkiye`ye taşınmak istiyorum………………………………... ___________**2**
3. Almanya`da kendimi evimde hissediyorum………………….……………. ___________**3**
4. Türkiye`yi genel olarak tatilde kaldığım zamanlardan tanıyorum………… ___________**4**
5. Türkiye`de iken kendimi sıkça yabancı hissediyorum…………………... ___________**5**
6. Hiç bir yere dahil değilim, ne Almanya`ya ne de Türkiye`ye………... ____________**6**
7. Hem Almanya`da hem de Türkiye`de evimdeyim………………………... ____________**7**

**35.b) Çocuk doktorunuzla çoğunlukla hangi dili konuşuyorsunuz?**

Türkçe **Ο** Almanca  **Ο** Tercüman aracılığıyla Almanca **Ο** Başka bir dil:_______________

1. **Son olarak size, çocuk doktorunuzdan çocuğunuzun hastalıklarıyla ilgili bilgi almamıza izin verip vermiyeceğinizi sormak istiyorum. Burada bizi, doktor son bir yılda çocuğunuzu ateşli iken muayene ettiğinde hangi sonuçlara varmış, bu ilgilendiriyor. Bunun için sizin yazılı izninize, çocuk doktorunuzun ismine, çocuğunuzun isim ve doğum tarihine ihtiyacımız var.**

**Ο**  Ret ediyorum

**Ο** Kabul ediyorum

| **Çağrı:** Sadece kabul edildiyse  **b) Çocuklarınızın isim ve soyisimleri?** | **c) Çocuklarınızın doğum tarihi?** | **Çocuk doktorunuz kimdir?** |
| --- | --- | --- |
|  |  |  |

- **B İ T T İ -**

| **Çağrı:** Lütfen doldurun   1. **Soru sorulan annenin Almanca bilgisini nasıl değerlendiriyor sunuz?** |
| --- |

Pek iyi **Ο** İyi **Ο** Orta **Ο** Yeterli **Ο** Yetersiz **Ο**  Hiç Almanca bilmiyor **Ο**
